# Effects of different irrigation amounts on soil structure in newly cultivated land on the Loess Plateau

**DOI:** 10.1371/journal.pone.0323065

**Published:** 2025-05-08

**Authors:** Hongyi Pan, Qize Song, Luyao Yi, Bo Li, Rutian Bi

**Affiliations:** 1 College of Resources and Environment, Shanxi Agricultural University, Taigu, China; 2 Datong Daylily Industrial Development Research Institute, Datong, China; Rodale Institute, UNITED STATES OF AMERICA

## Abstract

It is imperative to promote water-saving irrigation technology and develop newly cultivated land in the Loess Plateau. This study focused on the interaction between roots and soil to examine the effects of different amounts of irrigation on soil structure of newly cultivated land. Herein, five irrigation levels were set, i.e., sufficient irrigation (W100), mild deficit irrigation (W80), moderate deficit irrigation (W60), severe deficit irrigation (W40), and rain-fed (RF). Physical properties and structural stability indexes of the rhizosphere soil were measured, and their relationship with plant root morphology were analyzed. The results showed that the soil structure under the high irrigation amount group (W80 and W100) was relatively stable. The average particle density of soil in each plot decreased significantly after the experiment, while the soil total porosity remained unchanged in W80 and W100 treatments. The proportion of large aggregates, the mean weight diameter, and the geometric mean diameter of soil significantly reduced in the low irrigation amount group (RF, W40, and W60). In contrast, the W100 and W80 treatments inhibited the decline in soil aggregate stability. Change in the generalized soil structure index (GSSI) and soil three-phase structure distance (STPSD) of W100 and W80 treatments were not significant, before and after the experiment. These results suggested that the soil in newly cultivated land treated with sufficient and mild deficit irrigation was closer to the ideal state for crops growing. Path analysis identified the average soil moisture content had the greatest negative effect on STPSD primarily through the root length, root surface area, and root dry weight. In conclusion, irrigation amount occupies a dominant position among all factors influencing soil structure considered in the study. And the mild deficit irrigation is suitable for agricultural cultivation in the Loess Plateau area, from the soil structure protection and water-saving perspectives.

## 1. Introduction

Soil is a crucial component of terrestrial ecosystems and important for agricultural productivity [1,2]. The quality of farmland soil structure directly affects the growth and yield of crops [3]. Poor soil structure impedes water and nutrient absorption of crops, resulting in yield reduction [4,5]. Situated on the eastern edge of the Loess Plateau, Shanxi Province is characterized by fragmented terrain. Cultivated land accounts for only 27.1% of the province's total land area, with two-thirds being low-yield fields, which seriously restricts the healthy development of local agriculture. Therefore, it is urgent to rationally develop new reclamation land, enhance reserve cultivated land resources, and improve the soil quality of cultivated land [6].

Compared to mature farmland, newly reclaimed croplands often exhibit inherent limitations including low soil fertility, lack of biological species, and particularly the poor structure that hinders agriculture production [7–9]. As a basic soil property, soil structure is typically assessed through bulk density, particle density, porosity, three soil phases, and water stability aggregates [10]. Soil structure is influenced by a variety of factors, including climatic conditions [11,12], tillage practices [13,14], fertilizers [15,16], and the addition of soil amendments [17]. Optimal soil structure can promote crop growth, enhancing both agricultural yield and efficiency.

Shanxi Province is located in arid and semi-arid areas, with less precipitation and severe water resource scarcity (per capita availability equal to one-seventh of the national average). Therefore, it is urgent to develop water-saving agriculture. As a pivotal practice in water-saving agriculture, irrigation management exerts a direct impact on the farmland soil structure [18,19]. Enhanced irrigation amounts increase the liquid and gaseous-phase volume fractions, and reduces the solid-phase volume fraction, hence improving the structural stability of soil and the development of crops. Furthermore, a large irrigation quota can increase the structural stability of soil aggregates, inhibit soil compaction, and improve the infiltration capacity of topsoil [20]. Notably, experimental trials in tomato cultivation systems reveal that high irrigation increases total soil porosity and soil colloidal content, thereby improving soil structure [21]. Some scholars have proved that the most direct way to improve the soil quality of newly cultivated land was increasing irrigation amount, which can not only reduce soil bulk density but also increase the total soil porosity [22,23]. Recent studies integrate intercropping systems with optimized irrigation regimes, to improve soil quality in the arid zones of Northwestern China [24,25]. Meanwhile, irrigation amount is an important factor that affects root growth and spatial distribution of crops [26,27]. Then plant roots can improve soil structure by staggering, interspersing, and root-soil bonding. Generally, root growth can influence the formation of large and medium aggregates in variable soils [28–30], increase the mean weight and geometric mean diameters of soil aggregates, as well as soil porosity [31–33]. A suitable soil structure is conducive to root growth, and the two promote each other [34]. Therefore, irrigation management can reshape the soil structure through direct or indirect root-soil interaction action [35,36].

Overall, current research on soil structure optimization by irrigation predominantly focuses on wastewater irrigation [37], and adding soil conditioners [38,39]. However, few investigations focused on the influence of irrigation amount on soil structure, specifically via soil-root interactions. In addition, prior studies have primarily targeted mature cultivated land [40–42], with systematic analyses on newly cultivated land remaining scarce. To address these gaps, this study conducted a two-year greenhouse experiment combining rainwater harvesting and limited supply irrigation in the Loess Plateau. Through systematic investigation of irrigation amounts, we quantified their impacts on soil physical properties and structural stability of soil in newly cultivated land. These findings elucidate the irrigation-mediated ped structural evolution in newly cultivated land, providing a reference for the sustainable development of local agriculture.

## 2. Materials and methods

### 2.1 Study area

The experiment was conducted at the Shou-Yang Dryland Agroecosystem National Field Scientific Observation and Research Station, during June to November 2022 (first planting) and May to September 2023 (second planting). The station is located in Jingshang Township, Shou-yang County, Shanxi Province, China (37°45′N, 113°12′E, 1202 m a.s.l.), with a semi-humid continental climate. The site features an annual average temperature of 7.4 °C, multi-year averages of 474.5 mm precipitation, and 1673.0 mm evaporation [43]. Situated on the Loess Plateau, the experimental plots contained virgin brown soils that had remained undisturbed by agricultural or silvicultural activities for over two decades. The basic physico-chemical properties of the rhizosphere soil (0–60 cm) under different treatments before the start of the experiment are shown in Table 1. In this experiment, the soil texture was classified according to international standards and judged to be weakly alkaline sandy loam [44].

**Table 1:** Basic physicochemical properties of rhizosphere soils (0–60 cm depth) of different treatments.

Treatment	Organic matter/(g·kg^ − 1^)	pH	Mechanical composition/%		
**Clay** **(<0.002 mm)**	**Silt** **(0.002–0.02 mm)**	**Sand** **(0.020–2.0 mm)**
RF	7.75	8.36	8.76	27.79	63.44
W40	8.51	8.46	12.30	23.06	64.64
W60	9.59	8.43	9.95	25.57	64.48
W80	10.21	8.38	10.17	27.97	61.86
W100	8.13	8.37	10.80	25.27	63.93

### 2.2 Experimental design

#### 2.2.1 Crop management.

*Solanum lycopersicum* cv. “Kaide” was employed as the model crop in this study. Before the experiment, all the plots were leveled and rotary-tilled, and fermented ovine manure was applied as base fertilizer. A 0.08 mm transparent polyethylene mulch was laid on the soil surface for evaporation suppression and weed control. Four-leaf-stage tomato seedlings were transplanted in plots along a north-south oriented. They were arranged in wide-narrow rows with a spacing of 70 cm and 50 cm alternated, and plant spacing of 50 cm, achieving 3.3 plants/m^2^ density. The drip irrigation system featured a single-pipe single-row arrangement, that is, one drip belt that irrigated one row of crops. All plots were fully irrigated one week pre-transplanting (planting water) and repeated on transplanting day (recovery water), to ensure the survival of seedlings. Moreover, all crops received identical nutrient management. A total of six times top dressings were applied during the tomato growth period, mainly including water-soluble blended compound fertilizer, magnesium sulfate potassium fertilizer, and diamine sulfate. All the fertilizers were mixed evenly with irrigation water. The area of each plot (10 × 8.5 m) and agronomic measures (such as pollination, hanging vines, topping, insect repellency, and weed removal) were the same.

#### 2.2.2 Experimental design and management.

Five treatments with different irrigation amounts were implemented, that is, sufficient irrigation (W100), mild deficit irrigation (W80), moderate deficit irrigation (W60), severe deficit irrigation (W40), and rain-fed (RF). Soil moisture content was continuously monitored using the Shang Monitor (Insentek Co. Ltd., China) installed at plot centers. Meanwhile, the gravimetric method was conducted through oven-drying to calibrate the values of soil moisture content periodically. Sufficient irrigation treatment (W100) was set as a bench. If the average soil moisture content within a planned wetting layer (0–40 cm depth) fell below its field capacity (FC), supplemental irrigation was triggered to maintain soil moisture at FC-level The irrigation amount of treatment W40, W60, and W80 per application was 40%, 60% and 80% of treatment W100, respectively. Irrigation frequency (5–7 day intervals) and schedule were the same for treatment W40-W100. However, treatment RF exclusively utilized natural precipitation harvested. The rainwater collection system is composed of collection surfaces (greenhouse cover film), catchment troughs, water tanks, and water pipelines. The rainwater harvested was evenly distributed to all five plots after each rainfall event. In other words, treatment W40-W100 received combined normal irrigation and harvested rainwater inputs, while treatment RF depended solely on precipitation. To avoid the soil moisture content of any plot exceeding FC thresholds, surplus rainwater is stored in a separate tank for use subsequently. This dual water management protocol ensured: (1) equivalent precipitation inputs across all treatments, and (2) form distinct gradients of irrigation amount that strictly adhere to designated thresholds.

The actual irrigation amounts across treatments are shown in Table 2. Relative to the treatment W100, the irrigation amounts of the RF, W40, W60, and W80 treatments in 2022 were 55.96%, 64.61%, 79.43%, and 91.63%, respectively. In 2023, these ratios shifted to 50.90% (RF), 72.04% (W40), 83.32% (W60), and 90.64% (W80), respectively. The highest average soil moisture content within the rhizosphere soil (0–60 cm) was found in the W80 and W100 treatments for 2022 and 2023, respectively. Conversely, treatment W60 consistently exhibited the lowest average soil moisture levels across both growing seasons.

**Table 2:** Total irrigation amount (m^3^) and average soil water content (%) with rhizosphere zones (0–60 cm depth) during the whole growth period of tomato plants for different treatments.

Treatment	2022 (First planting)			2023 (Second planting)		
**Number of** **irrigation**	**Irrigation** **amount (m**^**3**^)	**Average soil moisture content** **(%)**	**Number of** **irrigation**	**Irrigation** **amount (m**^**3**^)	**Average soil moisture content (%)**
RF	10	13.99 (4.36)	13.07	12	15.39 (2.94)	12.20
W40	12	15.48 (4.36)	11.42	17	20.74 (3.12)	10.67
W60	12	18.03 (4.36)	10.27	17	23.40 (3.02)	9.65
W80	12	20.13 (4.36)	13.53	17	25.20 (3.03)	12.34
W100	12	21.37 (4.16)	12.66	17	27.47 (3.01)	14.82

**Note:** Values in parentheses refer to the sum of the planting and recovery water amounts.

### 2.3 Sample collection and measurement

#### 2.3.1 Soil samples.

The test pit was excavated in the middle of each plot, and soil samples from the 10, 20, 30, 40, and 60 cm depth were collected, comprising a total of three replicates. Then the soil samples were brought to the laboratory to evaluate their physical properties. Soil bulk density, soil particle density, water-stable aggregates, total soil porosity, and soil three-phase volume fraction were measured immediately according to the literature [45]. The soil was sampled three times in this trial, that is, before the experiment (February 2022), the first planting was harvested (February 2023), and the second planting was harvested (September 2023). Owing to the coronavirus pandemic, the soil sample scheduled for November 2022 at the harvest of the first planting was postponed to February 2023. While the experimental plots were not disturbed before the delayed sampling. Therefore, it was assumed that the soil was maintained in its original state to a certain extent after the first planting.

Soil bulk density (BD, g·cm^−3^) was determined using the ring knife method. The ring knife filled with undisturbed soil was weighed and placed in an oven at 105 °C to constant weight. The soil BD was calculated as follows:


$$BD=\frac{W_{1}-W_{0}}{V}$$ (1)

Where W_1_ is the hollow ring knife plus drying soil quality (g); W_0_ is the mass of the hollow ring cutter (g); and V is the ring knife volume (100 cm^3^).

Soil particle density (PD, g·cm^−3^) was determined using the pycnometer method. The relationship between the total mass of the pycnometer and the water temperature was plotted. Then, 15 g of dried soil sample and an appropriate amount of pure water were placed into the pycnometer. It was boiled in a sand bath for 30 min, filled with pure water, and moved into a constant-temperature water tank to measure the water temperature in the bottle and the total mass of the bottle, water, and soil. The soil PD was calculated as follows:


$$PD=\frac{\rho_{W}\times m_{0}}{m_{0}+m_{1}-m_{2}}$$ (2)

Where, m_0_ is the quality of the dried soil sample, g; ρ_W_ is the water density, taken as 9.98 g·cm^−3^; m_1_ is the total mass of the pycnometer and water at a specific temperature, g; m^2^ is the total mass of pycnometer, water, and soil at a specific temperature, g.

The total soil porosity (TP, %) is calculated by soil BD (g·cm^−3^) and soil PD (g·cm^−3^) as follows:


$$\begin{array}{c} \text{TP}=\left(1-\text{BD}/\text{PD}\right)\times 100\;\;\;\\ \;\;\; \end{array}$$ (3)

Water-stable aggregates were identified using the wet sieve method. First, 50 g of dried soil samples were prepared according to the mass fraction of each particle size obtained by the dry sieve method. They were then placed in the sieve of a TPF-100 aggregate analyzer (Zhejiang Top Yun-Nong Technology Co., Ltd., China) stacked in the order of 2 mm, 0.25 mm, and 0.053 mm pore size. The sieve containing the soil sample was soaked in water for 5 min and shaken for 5 min. After the shaking was completed, the residual soil samples in the sieve of each particle size were rinsed into an aluminum box and placed in an oven. Here they were dried to a constant weight at 60 °C. The mass of the dried soil for each particle size was measured and its mass fraction was calculated as the content of soil water-stable aggregates for each particle size.

The content of water-stable aggregates greater than 0.25 mm (M_R0.25_), mean weight diameter (MWD), and geometric mean diameter (GMD) were selected to evaluate the stability of soil aggregates [46]. The higher the index value, the more stable the soil aggregates. These indexes were calculated using the following formulas:


$$w_{i}=\frac{m_{i}}{M_{i}}\times 100\;$$ (4)

$$\begin{array}{c} M_{R0.25}=\frac{M_{R0.25}}{M_{T}}\times 100 \end{array}$$ (5)

$$MWD=\sum_{i=1}^{n}x_{i}\times W_{i}$$ (6)

$$GMD=exp\left[\frac{\sum_{i=1}^{n}W_{i}\times ln\;\bar{x_{i}}}{\sum_{i=1}^{n}W_{i}}\right]$$ (7)

Where W_i_ is the ith particle size aggregate mass fraction, %; m_i_ and M_i_ was the aggregate mass of the ith particle size and the overall mass of the aggregate, respectively, g. M_R0.25_ is the content of aggregates with a particle size greater than 0.25 mm, g; M_T_ is the total mass of the aggregate, g; $\;{\bar{x}}_{i}$ is the average value of the ith particle size range, mm.

Soil structural analysis quantified three-phase volumetric proportions, that is the solid phase volume fraction (XS), liquid phase volume fraction (XL), and gaseous phase volume fraction (XG). The soil three-phase structural distance (STPSD) represents the structural distance between the actual soil and the ideal soil three-phase state (solid: liquid: gaseous = 50: 25: 25) [47]. The smaller the value, the closer the soil is to the ideal level state. The generalized soil structure index (GSSI) is a production function representing the three-phase soil input and soil structure output, reflecting dynamic changes in soil structure. The closer the value is to 100, the closer the soil structure is to the ideal state for crop growth and the more stable the soil structure [48]. These indicators are calculated as follows:


$$X_{S}\left(\;\;\;\;\;\;\;\right)=100-TP$$ (8)

$$X_{L}\left(\;\right)=\theta_{r}\times BD$$ (9)

$$X_{G}\left(\%\right)=TP-X_{L}$$ (10)

$$STPSD=\sqrt{{\left(X_{S}-50\right)}^{2}+{\left(X_{L}-25\right)}^{2}+{\left(X_{G}-25\right)}^{2}}.$$ (11)

$$GSSI={\left[\left(X_{S}-25\right)X_{L}X_{G}\right]}^{0.4769}$$ (12)

Where, θr is the moisture content of soil mass, %; BD is the average bulk density of the soil; TP is the total porosity of the soil; XS is the volume fraction of soil solid phase; XL is the volume fraction of soil liquid phase; XG is the volume fraction of soil gaseous phase.

#### 2.3.2 Plant roots samples.

After the fruit was harvested, three representative tomato plants were selected from each plot for root sampling (in November 2022 and September 2023). With the plant's main stem as the center, seven sampling points were evenly arranged within a radius of 30 cm. The plant roots were collected using a root drill (10 cm inner diameter, 1020 cm^3^ volume) to a depth of 60 cm at 10 cm intervals. The root samples were carefully selected from the soil imminently, then washed and dried up. The fresh weight of the root was determined by electronic balance (Shimadzu, Ltd., Japan). After that, the morphological indicators of the root: (root length, root surface area, root diameter, and root volume) were obtained using a Performance V800 photo scanner (Epson Co., Ltd., Japan) and Win Rhizo root analysis software (Regent Instruments Inc., Canada). Then the roots were placed in an oven, dried at 105 °C for 30 min, and dried at 70 °C to constant weight. The dry weight of the roots was then recorded.

### 2.4 Data processing and analysis

Raw data were processed using Microsoft Excel 2021 (Microsoft Corporation, United States). Pearson correlation analysis and path analysis were performed using IBM SPSS 26.0 (IBM SPSS, Inc., United States). For the correlation analysis, the Shapiro-Wilk test is first used to determine the normality of the test data, and the data that does not satisfy the normal distribution is adjusted. Secondly, the absolute value of the Pearson product-moment correlation coefficient between different variable data is judged. If the absolute value is greater than or equal to 0.4, the two variables are considered to be related. In the path analysis, the causal relationship model between variables is constructed. The least square method or Maximum likelihood estimation method is used to estimate the path coefficient to form the total effect of the independent variable on the dependent variable, which is decomposed into direct effect and indirect effect. The significance test levels of the above statistical analysis methods and correlation coefficients were all at P < 0.05 [49,50]. Drawing was done using Origin 2022Pro software (Origin Lab, Inc., United States).

## 3. Results and analysis

### 3.1 Effects of different irrigation amounts on soil BD, PD, and TP

The average BD of the rhizosphere soil (0–60 cm) in the RF, W40, W60, and W80 treatments increased (Table 3) at the second planting was harvested compared with that before the experiment (February 2022), and the W40 treatment increased the most (5.34%). In contrast, the average BD of W100 treatment decreased by 3.67%. However, none of these changes were statistically significant. Furthermore, there was no significant difference in the average BD of the rhizosphere soil between the different treatments in the same sampling period.

**Table 3:** Changes in average BD, PD, and TP of rhizosphere soil (0–60 cm) under different treatments before and after the experiment.

	Treatment	February 2022	February 2023	September 2023
**Soil BD (g·cm**^** − 3**^)	RF	1.38 ± 0.07 aA	1.38 ± 0.09 aA	1.41 ± 0.16 aA
	W40	1.31 ± 0.05 aA	1.36 ± 0.06 aA	1.38 ± 0.04 aA
	W60	1.36 ± 0.05 aA	1.39 ± 0.03 aA	1.37 ± 0.06 aA
	W80	1.37 ± 0.07 aA	1.42 ± 0.14 aA	1.38 ± 0.07 aA
	W100	1.36 ± 0.06 aA	1.34 ± 0.06 aA	1.31 ± 0.06 aA
**Soil PD (g·cm**^** − 3**^)	RF	2.49 ± 0.04 aA	2.51 ± 0.04 bA	2.36 ± 0.07 aB
	W40	2.48 ± 0.04 aA	2.49 ± 0.04 bA	2.37 ± 0.04 aB
	W60	2.46 ± 0.03 aA	2.53 ± 0.03 bA	2.35 ± 0.09 aB
	W80	2.50 ± 0.03 aA	2.59 ± 0.02 aA	2.39 ± 0.08 aB
	W100	2.49 ± 0.05 aA	2.50 ± 0.04 bA	2.38 ± 0.06 aB
**Soil TP (%)**	RF	44.66 ± 2.88 aA	45.03 ± 3.45 aA	40.28 ± 5.89 bB
	W40	46.99 ± 1.18 aA	45.46 ± 1.71 aA	41.6 ± 1.65 abB
	W60	44.74 ± 2.11 aA	45.07 ± 1.13 aA	41.83 ± 2.06 abB
	W80	45.20 ± 2.28 aA	45.08 ± 4.87 aA	42.68 ± 1.97 abA
	W100	45.56 ± 2.88 aA	46.50 ± 2.07 aA	45.15 ± 2.62 aA

**Note:** The data are all mean ± standard deviations. Different lowercase letters in the same column indicate significant differences between treatments in the same period (P < 0.05). Different uppercase letters in the same row indicate significant differences in treatments among different periods in the same treatments (P < 0.05).

The average soil PD for different treatments changed significantly before and after the experiment. The relative size relationships were February of 2023 > February of 2022 > September 2023. Compared with that before the experiment, the average PD of the rhizosphere soil decreased significantly by 4.48% (RF), 4.44% (W40), 4.47% (W60), 4.40% (W80), and 4.42% (W100) at the second planting was harvested, respectively. In February 2022 and September 2023, there were no significant differences in the average PD among different treatments. In February 2023, the average PD of the W80 treatment (2.59 g cm^−3^) was significantly higher than that in the other treatments.

The non-significant change was found in the TP of rhizosphere soils for all treatments in February 2023 compared with that before the experiment (Table 3). However, by the end of the experiment in September 2023, the TP in the RF, W40, and W60 treatments was significantly reduced by 9.81%, 11.47%, and 6.50%, respectively. Compared with the low irrigation amount treatments, the TP of the W80 and W100 treatments were stabled at a relatively high level. The TP of the rhizosphere soil was significantly different across treatments only at the last sampling.

### 3.2 Effects of different irrigation amounts on soil water-stable aggregates

**Fig 1:**
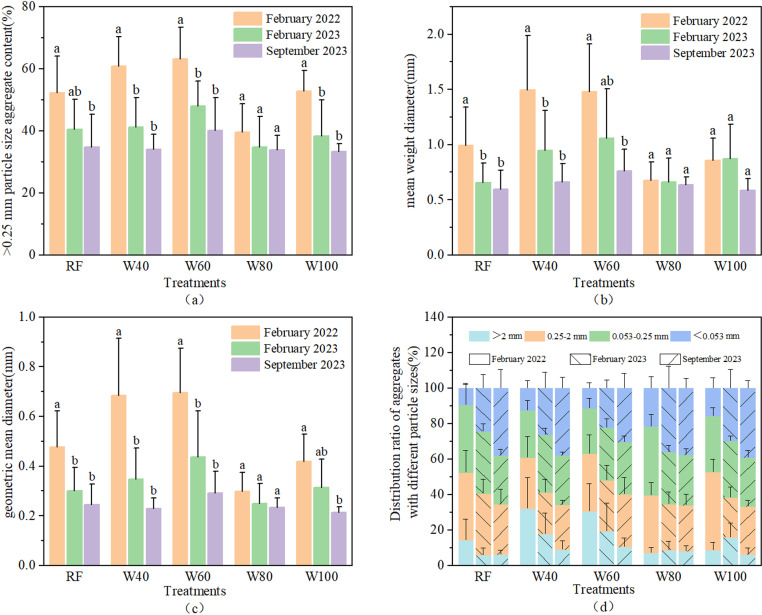
Changes in soil aggregate-related indexes before and after the experiment **(a)** >0.25 mm particle size aggregate content; **(b)** MWD of soil aggregates; **(c)** GMD of soil aggregates; **(d)** Distribution ratio of aggregates with different particle sizes).

Changes in soil water stability under different irrigation levels are shown in Fig 1. The M_R0.25_, the MWD, and the GMD of soil macroaggregates have consistent regularity in all treatments. Compared with that before trial, the above indicators decreased significantly after the experiment. In contrast, the content of the small aggregates in each treatment increased by the end of the experiment.

In February 2023, the M_R0.25_ of W40, W60, and W100 treatments, decreased from 52.29%, 60.83%, and 63.10% before trial to 40.45%, 41.19%, and 48.03%, respectively (Fig 1a). At the end of the experiment (September 2023), the M_R0.25_ of all treatments except the W80 was significantly lower than that in February 2022. The reduction of the W40 treatment was the largest (43.87%), and the RF treatment was the smallest (33.54%). The MWD of the RF and W40 treatments in February 2023 was significantly reduced than that before the experiment, and no significant change was found in the other treatments (Fig 1b). However, the MWD in September 2023 decreased by 40.04% (RF), 55.82% (W40), 48.58% (W60), 6.19% (W80), and 31.93% (W100) significantly, compared to baseline. The W80 and W100 treatments could alleviate the decline of soil aggregate stability to a certain extent. The GMD reflects the stability of soil aggregates and their resistance to erosion. The higher the GMD value, the more stable the soil aggregates, and the stronger the erosion resistance. Compared to that before the experiment, the GMD of all treatments showed a downward trend after the trial (September 2023, Fig 1c). These differences have statistical significance, except for the W80 treatment. While, the W40 treatment showed the largest decrease, reaching 66.37%. Fig 1d shows the particle size distribution of the soil aggregates under different treatments before and after the experiment. At the end of the trial, the content of aggregates with particle sizes greater than 2 mm in the W40 and W60 treatments decreased from 32.14% and 30.54% before the test to 8.98% and 10.62%, respectively. In contrast, the content of aggregates with particle sizes greater than 2 mm in the W80 treatment increased from 6.98% to 7.95%, representing an increase of 14.20%. The content of aggregates with particle sizes less than 0.053 mm in each treatment increased significantly compared to that before the experiment, with increases of 295.20% (RF), 197.41% (W40), 163.75% (W60), 74.14% (W80), and 148.60% (W100).

### 3.3 Effects of different irrigation amounts on soil three-phase ratio

**Fig 2:**
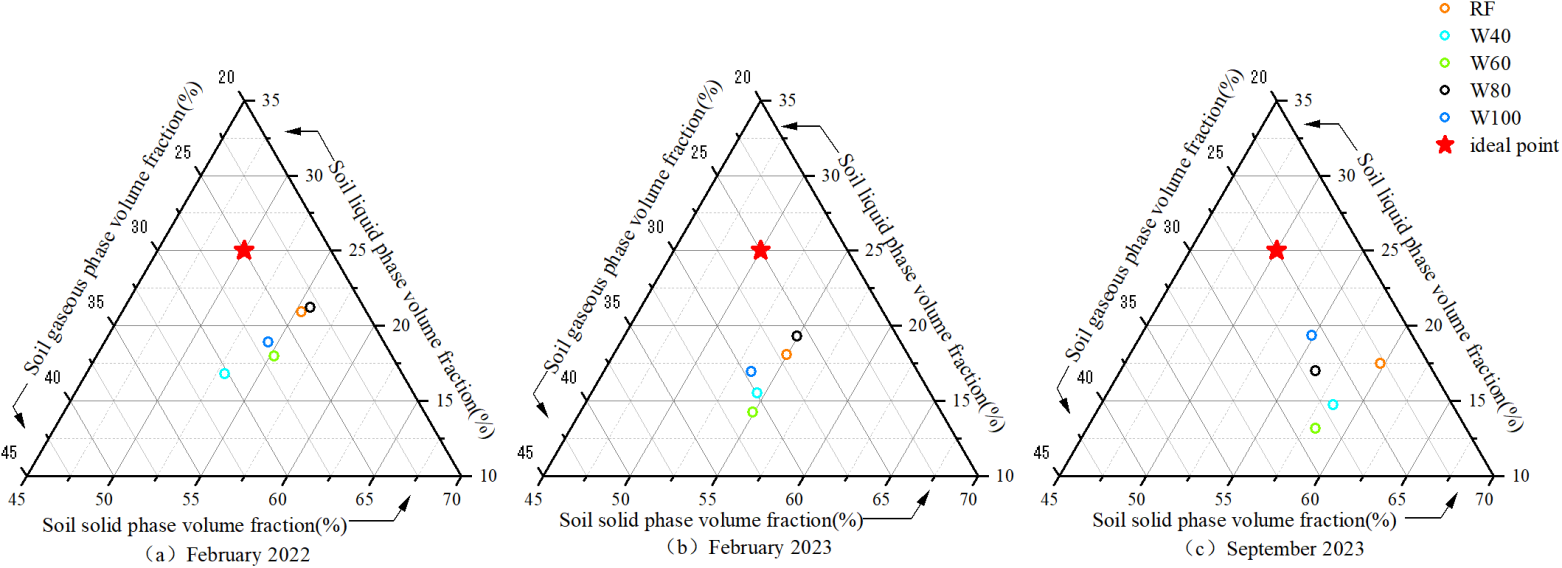
Relationship between soil three phases and ideal points in different treatments before and after the experiment.

The ideal point refers to the ideal three-phase state (volume for solid, liquid, and gaseous are: 50: 25: 25) of the soil that is most suitable for crop growth. As shown in Fig 2, in February 2022 and 2023, the soil three-phase ratio of the W80 treatment was closer to the ideal point, with 55.7: 21.2: 23.1 and 54.9: 19.3: 25.8, respectively. In September 2023, the soil three-phase of the W100 treatment was closer to the ideal point with a ratio of 54.9: 19.4: 25.8. After the experiment, the soil solid phase volume fraction of each treatment increased compared with that before the trial. The W40 treatment changed the most, from 53.01% to 58.40% with, an increase of 10.17%. The soil liquid phase volume fraction of the W100 treatment in September 2023 increased compared with that before the experiment. However, the other treatments had the opposite effect (reduction of 16.29–29.72%). The soil gaseous volume fractions in the RF, W60, W80, and W100 treatments decreased after the experiment, and the W40 treatment showed the opposite trend. However, the differences across all treatments were not significant.

To determine the distance between the three phases ratio of the actual soil and the ideal point, the STPSD and GSSI were calculated (Table 4). Compared with pre-experiment values, the STPSD in all treatments showed an upward trend by the end of the trial, except for the W100 treatment. The STPSD of the RF, W40, and W60 treatments showed significant increases of 107.48%, 33.27%, and 59.07%, respectively. The ranking of the STPSD across treatments changed from W80 > RF > W60 > W100 > W40 (before the experiment) to W100 > W80 > W40 > W60 > RF(after the experiment). GSSI is an indicator where values closer to 100 represent more stable soil structures and better suitability for crop growth. Compared with that before the experiment, the GSSI in September 2023 significantly decreased by 9.88%, 8.34%, and 7.07% for RF, W40, and W60 treatments, respectively. In contrast, the GSSI of the W100 treatment slightly increased by 1.24% (non-significant). Meanwhile, the W80 treatment also maintained statistically unchanged GSSI levels before and after the experiment. These results suggest that sufficient and mild deficit irrigation better preserves soil structural stability, maintaining conditions closer to the ideal state for crop growth.

**Table 4:** Changes in soil three-phase structure distance (STPSD) and generalized soil structure index (GSSI) before and after the experiment.

	Treatment	February 2022	February 2023	September 2023
**Soil three-phase structure distance**	RF	8.15 ± 3.57 aB	10.08 ± 2.52 aB	16.91 ± 4.69 aA
	W40	10.25 ± 3.41 aB	12.05 ± 1.54 aAB	13.66 ± 0.88 aA
	W60	9.43 ± 2.02 aB	13.26 ± 1.82 aA	15.00 ± 2.80 aA
	W80	7.48 ± 2.53 aA	10.89 ± 3.56 aA	11.25 ± 2.51 aA
	W100	9.54 ± 2.76 aA	10.26 ± 3.21 aA	8.26 ± 1.83 aA
**Generalized soil structure index**	RF	97.03 ± 1.95 aA	95.87 ± 2.07 aA	87.44 ± 6.06 cB
	W40	95.01 ± 3.01 abA	93.59 ± 1.79 aA	87.09 ± 1.26 bcB
	W60	96.23 ± 1.65 abA	91.88 ± 2.35 aB	89.43 ± 3.82 cB
	W80	97.79 ± 1.28 bcA	95.22 ± 2.84 abA	94.48 ± 3.01 abA
	W100	96.06 ± 1.85 cA	95.21 ± 2.60 bA	97.26 ± 0.97 aA

**Note:** The data are all mean ± standard deviations. Different lowercase letters in the same column indicate significant differences between treatments in the same period (P < 0.05). Different uppercase letters in the same row indicate significant differences among different periods in the same treatment (P < 0.05).

### 3.4 Correlation between root morphology and soil structure indexes

**Fig 3:**
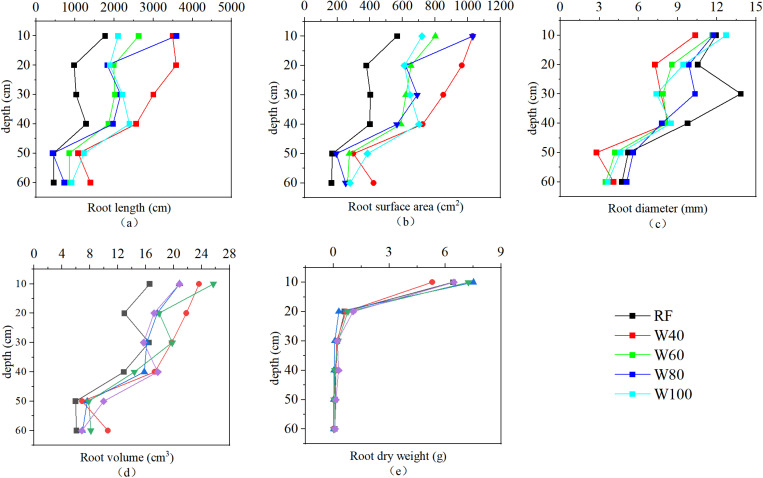
Changes in root morphology indexes and root dry weight of different treatments in November 2022 (First planting).

**Fig 4:**
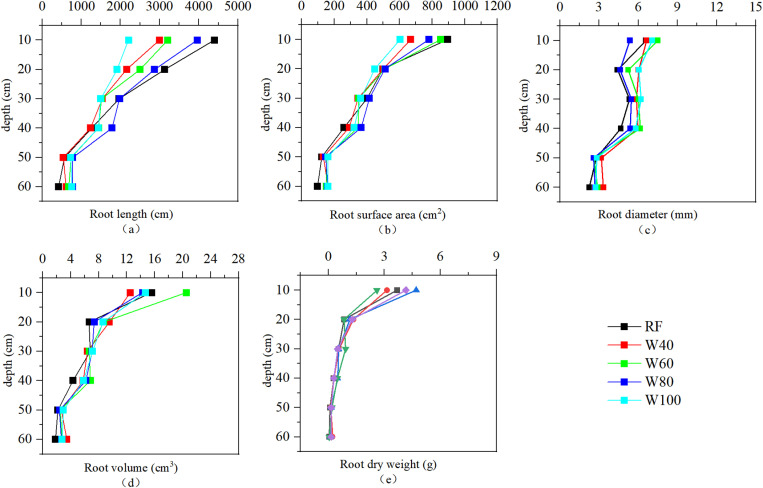
Changes in root morphology indexes and root dry weight of different treatments in September 2023 (Second planting).

As shown in Fig 3 and 4, the roots of tomato plants were mainly distributed in shallow soils (0–40 cm depth). With increasing soil depth, the morphological indexes and dry weight of the plant roots under different treatments generally showed a downward trend. The length, surface area, diameter, and volume of the roots began to decrease significantly at a depth of 40–50 cm. Meanwhile, an abrupt reduction in root dry weight was observed at a depth of 20 cm. At the first planting was harvested, tomato roots in the W40 treatment exhibited the largest total length and total surface area, whereas the RF treatment showed the smallest values for both parameters. In contrast, the average root diameter in the RF treatment was the largest (9.34 mm), while the W40 treatment showed the smallest value (6.75 mm). The root dry weight in the W80 treatment was the highest (8.36 g) in shallow soil (0–40 cm) and the lowest value (6.29 g) was found in the W40 treatment. At the harvest of the second planting, the total root length and total root surface area in the W80 treatment were the largest. The ranking of these parameters across treatments was: W80 > RF > W60 > W40 > W100. Furthermore, the root volume followed the order of W60 > W100 > W40 > W80 > RF, and the ranking of average root diameter was W40 > W100 > W60 > W80 > RF. The root dry weight of the W60 treatment was the highest (7.15 g), whereas that of the W80 treatment was the lowest (5.08 g). Overall, the root growth and development of sufficient irrigation treatment (W100) were generally weaker than those of deficit irrigation.

**Fig 5:**
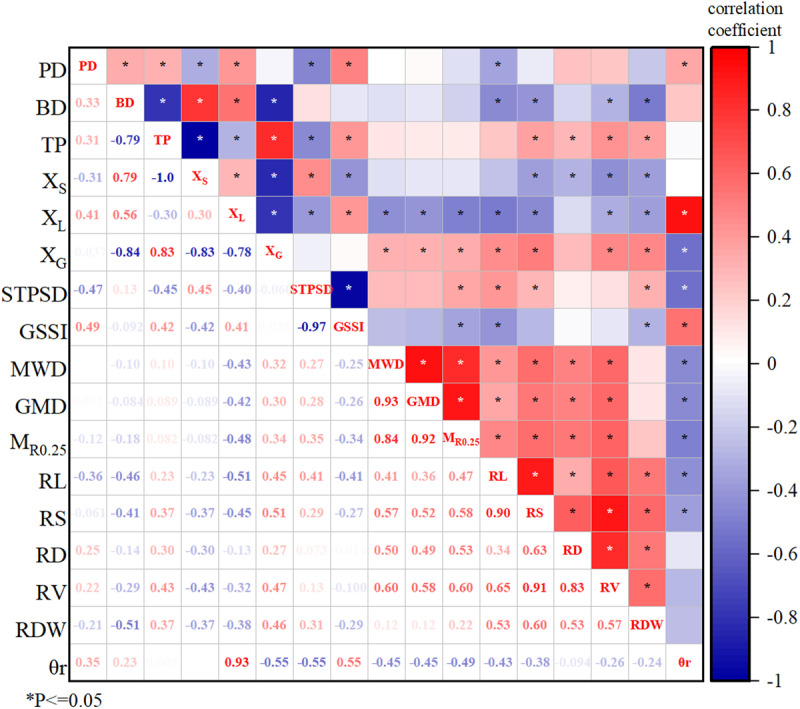
Thermal map of correlation between tomato root morphology and soil structure index. **Note:** M_R0.25_ is the mass fraction of soil macro aggregates, MWD is the mean weight diameter, GMD is the geometric mean diameter, BD is the average bulk density of the soil, PD is the soil particle density, TP is the total porosity of the soil, X_S_ is the soil solid phase volume fraction, X_L_ is the soil liquid phase volume fraction, X_G_ is the soil gaseous phase volume fraction, STPSD is the soil three-phase structure distance, GSSI is the generalized soil structure index, RL is the root length, RS is the root surface area, RD is the root diameter, RV is the root volume, RDW is the root dry weight, θr is the average soil moisture content within 0-60 cm depth during the whole growth season.

The soil structure indexes, root morphology indexes, and average soil moisture content measured at the root depth (0–60 cm) in the same period were selected for correlation analysis (Fig 5). Soil BD was significantly positively correlated with soil PD, soil solid phase volume fraction, and soil liquid phase volume fraction, whereas negatively correlated with soil TP and soil gaseous phase volume fraction. That is, an increase in soil PD means that the volume fractions of the solid and liquid phases increase and the volume fraction of the gaseous phase decreases. While decreasing the TP and air permeability of the soil increases the BD. GSSI was significantly positively correlated with soil PD, soil TP, and soil liquid phase volume fraction, with correlation coefficients of 0.49, 0.42, and 0.41, respectively. It was negatively correlated with soil solid phase volume fraction, STPSD, and macroaggregate content, with correlation coefficients of −0.42, −0.97, and −0.34, respectively. The larger the soil TP, soil PD, and soil liquid phase volume fraction, the smaller the soil solid phase volume fraction and aggregate content, and the closer the soil is to the ideal state for planting crops.

Root length was significantly positively correlated with soil gaseous phase volume fraction and water-stable aggregates, whereas negatively correlated with soil BD, soil PD, and soil liquid phase volume fraction. Root surface area and root volume were significantly positively correlated with soil TP, soil gaseous volume fraction, and aggregate stability. While negatively correlated with soil BD, liquid and solid phase volume fraction. Root dry weight was significantly positively correlated with soil TP and gaseous volume fraction and negatively correlated with soil BD, solid, and liquid phase volume fraction. STPSD was significantly and positively correlated with root length, root surface area, and root dry weight, with correlation coefficients of 0.41, 0.29, and 0.31, respectively. These results suggested that the growth and development of plant roots can improve the stability of soil water-stable aggregates, reduce soil BD, inhibit the decline in soil TP, and increase GSSI, thereby improving the stability of the soil structure. The average soil moisture content was significantly positively correlated with soil PD (r = 0.35), liquid phase volume fraction (r = 0.93), and GSSI (r = 0.55), whereas negatively correlated with soil gaseous phase volume fraction (r = -0.55), STPSD (r = -0.55), M_R0.25_ (r = -0.49), MWD (r = -0.45), GMD (r = -0.45), root length (r = -0.43), and root surface area (r = -0.38). The higher the average soil moisture content, the closer the soil structure is to an ideal state for crop growth.

### 3.5 Path analysis of soil three-phase structure distance (STPSD)

To further examine how the factors affecting soil structure, STPSD was used as an example for path analysis. According to the results of correlation analysis (Fig 5), nine indicators, were selected as the impact factors. The results of path analysis are shown in Table 5. Due to the problem of multicollinearity in the soil solid phase volume fraction and soil liquid phase volume fraction, these two factors were excluded from the analysis.

**Table 5:** Path analysis of soil three-phase structure distances (STPSD).

Function factor	Direct path coefficient	Indirect path coefficient								Correlation coefficient
		X1	X2	X3	X4	X5	X6	X7	Total	
X1	0.117		−0.194	−0.017	−0.189	0.025	−0.088	−0.126	−0.589	−0.472
X2	−0.619	0.037		0.012	0.12	−0.153	0.156	0.003	0.174	−0.445
X3	0.145	−0.014	−0.051		0.246	−0.240	0.092	0.176	0.208	0.353
X4	0.525	−0.042	−0.141	0.068		−0.373	0.219	0.153	−0.116	0.409
X5	−0.416	−0.007	−0.228	0.084	0.47		0.25	0.134	0.703	0.287
X6	0.417	−0.025	−0.231	0.032	0.276	−0.249		0.086	−0.111	0.306
X7	−0.356	0.041	0.006	−0.072	−0.225	0.156	−0.100		−0.194	−0.550

**Note:** X1 is the soil PD, X2 is the soil TP, X3 is the content of M_R0.25_, X4 is the root length, X5 is the root surface area, X6 is the root dry weight, and X7 is the average soil moisture content.

The absolute value of the direct path coefficient reflects the direct influence of the factor on the dependent variable. The direct effects of soil TP(X2), root length (X4), root dry weight (X6), root surface area (X5), average soil moisture content (X7), macroaggregate content (X3), and soil PD (X1) on STPSD gradually decreased. Among them, soil PD, macroaggregate content, root length, and root dry weight had direct positive effects on STPSD. Whereas, soil TP, root surface area, and average soil moisture content had direct negative effects on STPSD.

The magnitude of the indirect path coefficient reflects the degree to which one factor indirectly affects the dependent variable through other factors. Soil TP, large aggregate content, and root surface area had indirect positive effects on STPSD. Among them, the indirect effect of root surface area was the largest (0.703), and it was mainly affected by root length (0.470), root dry weight (0.250), and soil total porosity (−0.228). Soil PD, root length, root dry weight, and average soil moisture content had indirect negative effects on the STPSD. Among them, the effect of soil PD was the most obvious (−0.589), and STPSD was mainly affected by soil TP (−0.194), root length (−0.189), and average soil moisture content (−0.126).

Although soil PD had a direct positive effect on STPSD, it was significantly negatively correlated with STPSD under the indirect effects of soil TP, root length, and average soil moisture content. In contrast, the direct effect of the root surface area on STPSD was negative. However, it was significantly positively correlated with STPSD through the indirect effects of root length, root dry weight, and average soil moisture content. Soil TP had the largest direct negative effect and the smallest indirect positive effect on STPSD. Root length and dry weight had opposite effects. Although their indirect effects were negative, both of them were significantly and positively correlated with STPSD. The direct effects of soil macroaggregate content and average soil moisture content on the STPSD were consistent with their indirect effects. Among all factors, the average soil moisture content had the greatest effect (r = −0.550) on the STPSD, whereas the effect of the root surface area was minimal (r = 0.287).

## 4. Discussion

### 4.1 High irrigation amount can reduce the soil PD and maintain the soil BD and TP

At the second planting was harvested, the average BD of the rhizosphere soil in the deficit irrigation treatment increased compared to that before the experiment. Meanwhile, the sufficient irrigation treatment showed the opposite, although the difference was not significant (Table 3). This was consistent with the findings of Zhang et al. regarding the physical properties of soils in greenhouse [21].On the one hand, the soil compaction caused by frequent movement of artificial irrigation causes the gas in the soil to be removed and the soil bulk density to be reduced [51,52]. On the other hand, an increase in irrigation amount may lead to an increase in soil liquid phase volume fraction and cohesion, whereas a decrease in soil hardness [53]. This, in turn, reduces soil BD [54]. The growth and development of plant roots also affect soil BD. Strong crop roots can stabilize soil traits, and reduce the risk of soil compaction [55]. Similarly, we found that soil BD was negatively correlated with plant root-related indexes (Fig 5). The development under deficit irrigation levels (RF, W40, and W60 treatments) was generally better (Fig 3 and 4). However, the average soil BD increased slightly, indicating that the effect of crop roots on soil BD may be weaker than that of irrigation amount. Therefore, under the combined effect of irrigation amount and root development, there was no significant change in soil BD under the different treatments before and after the experiment. Khan et al. reached similar conclusions when they investigated the effects of the irrigation level on soil profile [56].

Soil PD is also an indicator used to evaluate soil structure. The lower the soil particle density, the greater the soil aeration and field capacity [57,58]. After the experiment, the average PD of the rhizosphere soil in all treatments decreased significantly compared with that before the experiment (Table 3). This is consistent with Badaou, who found that irrigation practices could reduce soil PD [59]. However, the correlation analysis showed that there was a significant positive correlation between soil PD and average soil moisture content. This indicated that the larger the irrigation amount, the smaller the decrease in soil PD. Changes in the soil PD may be related to plant root growth and development. We found a significant negative correlation between root length and soil PD (Fig 5). He et al. also suggested that the development of the root structure of pioneer plants could reduce the PD of clay-red soil [60]. Similar results were reported in a study on the root growth and the distribution of bananas [61].

At the second planting was harvested, the TP of the rhizosphere soil among different treatments decreased compared to that before the experiment. However, there was no significant difference between the sufficient and mild deficit irrigation treatments (Table 3). High irrigation amount slows the decline in soil TP. This may be related to changes in soil aggregates [62]. Higher irrigation volume could destroy the aggregate structure of the soil, causing macroaggregates to decompose into microaggregates. This, in turn, increases the soil porosity and TP [63]. Jin et al. found that increasing irrigation was beneficial in improving the TP of the soil. Plant root growth affects soil TP as well. Accompany the development of the root, the root tips can extend into the deep soil and develop more fully in the biological pores, increasing the soil TP [33]. We found that root surface area, root diameter, root volume, and root dry weight were positively correlated with soil TP (Fig 5). Well-developed roots were able to produce more macropores in the soil, increasing soil TP [64]. Wang also highlighted that there was a significant positive correlation between soil TP and crop roots, that is, the length, surface area, and volume. However, we found that although the root growth under the low irrigation treatment was generally better than that under sufficient irrigation, the soil TP decreased significantly (Table 3). This indicated that the effect of the irrigation amount on soil TP was dominant. Therefore, the TP of the soil treated with a high amount of irrigation was maintained at a high level.

### 4.2 High irrigation amount can inhibit the decline of water-stable aggregate structure stability

We found that the proportion of large aggregates (M_R0.25_), mean weight diameter (MWD) and geometric mean diameter (GMD) of water-stable aggregates decreased after the experiment (Fig 1). Wang et al. found that short-term planting could cause a decrease in the content of soil macroaggregates and an increase in the number of microaggregates. This may be caused by a decrease in soil organic matter content due to crop growth. This leads to the loss of cementation of soil macro aggregates and the formation of more small aggregates. However, there were no significant differences in the M_R0.25_, MWD, and GMD of W80 treatment, before and after the experiment (Fig 1). This indicates that high irrigation amount could inhibit the decline in structural stability of water-stable aggregates. Generally, irrigation can increase the internal friction angle of soil, thereby improving soil osmotic resistance and aggregate stability [54,65]. However, with an increase in irrigation amount, the water-filled distance between soil particles increases, resulting in soil expansion, weakening of van der Waals forces, and aggregates disintegrating [66]. The length, surface area, diameter, and volume of roots were significantly and positively correlated with the M_R0.25_, MWD, and GMD of soil macroaggregates (Fig 5), which is consistent with others [67]. The root growth under low irrigation amount treatments was generally better than those under sufficient irrigation. However, the decrease in water-stable aggregates was more pronounced after the experiment, indicating that the influence of irrigation amount may be dominant. Therefore, high irrigation amounts (W80 and W100 treatments) slowed down the downward trend of water-stable aggregates. However, the correlation analysis showed that the M_R0.25_, MWD, and GMD of soil macroaggregates were significantly negatively correlated with the average soil moisture content (Fig 5). This may be due to some other factors that affect the water-stable aggregates, such as root exudates, which were not considered in this study. The combined action of these factors negatively correlated the soil water-stable aggregates with the average soil moisture content.

### 4.3 The soil structure under the high irrigation amount treatment is closer to the ideal state

The STPSD was negatively correlated with soil PD, soil total porosity, and soil average moisture content (Fig 5). This means that with the soil TP increased, soil air permeability was enhanced, and the soil structure improved. Meanwhile, the larger the average soil moisture content, the closer the soil structure is to the ideal state. This is similar to the studies on flooding and intermittent irrigation [68]. Some evidence showed that increasing irrigation amount can increase the soil volume and viscosity, reduce the soil gaseous volume fraction [69], thus improve the three-phase structure of the soil [70] and bring the soil structure closer to the ideal state [47,71]. However, the M_R0.25_, MWD, and GMD of soil macroaggregates were positively correlated with the STPSD. In other words, the more stable the soil water-stable aggregates are, the farther the soil structure to the ideal point, which is abnormal. On the one hand, this might relate to the factors such as the microorganisms, which can affect soil water-stable aggregates but were not analyzed in this study. On the other hand, the M_R0.25_, MWD, and GMD of soil macroaggregates in RF, W40, and W60 treatments were higher than those of W80 and W100 treatments at the beginning of the experiment (February 2022,Fig 1). Therefore, the M_R0.25_, MWD, and GMD of soil macroaggregates and STPSD in W80 and W100 treatments were lower simultaneous after the experiment.

Furthermore, the changes in solid, liquid, and gaseous phase volume fraction of the soil indicate that the soil permeability decreased (Fig 2), compared to pre-experiment. Deng et al. noted that irrigation can increase the solid phase volume fraction of soil and decrease their liquid and gaseous phase volume fractions [72], which may be related to the compaction of farmland [73].

There was a significant positive correlation between root length, root surface area, root dry weight, and the STPSD (Fig 5). That is, the greater the STPSD when the plant roots grow vigorously. However, the average soil moisture content had a stronger effect on the STPSD than the root. Therefore, the STPSD of the high irrigation treatments (W80 and W100) was smaller, and their soil structure was closer to the ideal state. The root development of the high irrigation amount treatments may be weaker than that of other treatments, it can also achieve the effect of improving soil structure. This is consistent with the other's report [74].

### 4.4 Limitations of this study

Due to the impact of the COVID-19 pandemic, soil samples that were supposed to be obtained in November 2022 were delayed in February 2023. Postponed field collection exposed samples to extended environmental exposure, potentially subjecting them to freeze-thaw cycles and natural air-drying. This methodological deviation requires critical consideration, as Sun pointed out that freeze-thaw and air-drying increased soil porosity [75]. Concurrently, multiple studies have established that air drying has an impact on soil aggregates and bulk density [76–78]. Therefore, the structure of the soil sampled in February 2023 may have changed. In addition, the wilting of roots in the soil also affects the TP and aggregate of the soil [79,80]. These compound effects-environmental exposure and root decomposition may introduce confounding variables in the interpretation of soil structure.

This study focuses on the interaction between soil structure and plant root morphology under different irrigation amounts. However, soil structure was also affected by root exudates. Relevant reports have shown that root exudates mainly influence soil nutrients (total nitrogen, total phosphorus, total potassium) and soil microorganisms, thereby indirectly affecting soil structure [81,82]. Thus, the generalizability of this study might be limited, since the relationship between the physical properties of soil and root exudates was not considered. It would be beneficial to incorporate new indicators to further explore the factors that impact the soil structure of newly cultivated land on the Loess Plateau under different irrigation amounts.

## 5. Conclusion

The soil structure under the high irrigation amount treatments (W80 and W100) was relatively stable during the experimental period. After the experiment, the soil PD decreased significantly across all treatments compared with the pre-experiment. The soil TP remained statistically unchanged in W80 and W100 treatments, while the low irrigation amount groups (RF, W40, and W60) exhibited significant TP decreases of 6.5–11.5%. After the experiment, the M_R0.25_, MWD, and GMD of the soil macroaggregates decreased compared with the baseline, except for the W80 treatment. Path analysis showed that the average soil moisture content had the greatest negative influence on the STPSD primarily through the root length, root surface area, and root dry weight. This study demonstrated that the changes in soil structure were inseparable from the joint action of water, root, and soil, and irrigation amount occupied a dominant position among these factors. Furthermore, the GSSI of the low irrigation amount treatments was significantly lower than that before the experiment (STPSD was vice versa). These results suggested that the soil in newly cultivated land treated with sufficient and mild deficit irrigation was closer to the ideal state for crops growing. In conclusion, mild deficit irrigation (W80) is suitable for agricultural cultivation in the Loess Plateau area, considering soil structure protection and water-saving.

## Supporting information

S1 Table Basic physicochemical properties of rhizosphere soils (0–60 cm) of different treatments on the test location. (XLSX)

S2 Table Basic physical properties and structural indexes of soil after two planting phases. (XLSX)

S3 Table Root index data of tomato after two planting stages. (XLSX)
